# Extracellular vesicles carrying lactate dehydrogenase induce suicide in increased population density of ***Plasmodium falciparum in vitro***

**DOI:** 10.1038/s41598-019-41697-x

**Published:** 2019-03-25

**Authors:** Ricardo Correa, Lorena Coronado, Zuleima Caballero, Paula Faral, Carlos Robello, Carmenza Spadafora

**Affiliations:** 1 Center of Cellular and Molecular Biology of Diseases, Instituto de Investigaciones Científicas y Servicios de Alta Tecnología (INDICASAT AIP). City of Knowledge, Panama City, 0843-01103 Panama; 2 Department of Biotechnology, Acharya Nagarjuna University, Guntur, 522 510 A.P. India; 3 Institut Pasteur, Montevideo, Uruguay; 4 Sistema Nacional de Investigación, Secretaría Nacional de Ciencia, Tecnología e Innovación, Panama City, 0843-01103 Panama

## Abstract

Even with access to sufficient nutrients and atmosphere, *Plasmodium falciparum* can barely be cultured at maximum growth capacity *in vitro* conditions. Because of this behavior, it has been suggested that *P*. *falciparum* has self-regulatory mechanisms in response to density stress. Only recently has this process begun to be acknowledged and characteristics of a programmed cell death been assigned to the parasite at high parasitaemia *in vitro* cultures. In searching for death signals within the parasite community, we have found that extracellular vesicles (EVs) of *P*. *falciparum* from high parasitaemia cultures are able to induce programmed cell death processes in the population. A comparative proteomic analysis of EVs from low (EV_L_) and high (EV_H_) parasitaemia cultures was conducted, pointing to lactate dehydrogenase from *P*. *falciparum* (PfLDH) as the only parasite protein overexpressed in the later. Although the major function of *P*. *falciparum* lactate dehydrogenase (PfLDH) is the conversion of pyruvate to lactate, a key process in the production of energy in most living organisms, we investigated its possible role in the mechanism of parasite density control by intercellular signaling, given that PfLDH had already been listed as a component of extracellular vesicles of *P*. *falciparum*. In this study we present evidence of the EV-associated PfLDH regulation of parasite population by inducing apoptosis in highly parasitized cultures.

## Introduction

The improvement in control and treatment of malaria has reduced its worldwide impact in the last decade. However, the parasite that is the cause, *Plasmodium falciparum*, still results in approximately 429,000 deaths and 200 millions cases each year^1^. Experimental studies using *in vitro* culture have increased the understanding of the biology of *P*. *falciparum*, which powered the development of several strategies for malaria treatment. Interestingly, the parasitaemia growth of *P*. *falciparum* can reach a maximum multiplying factor of 8, far below a potential factor of 16^2^, even when the media culture is enriched with serum and nutrients and the apparent availability of uninfected erythrocytes is large. Based on this behavior, it has been suggested that *P*. *falciparum* uses self-regulation mechanisms in response to density stress^3^. This process in *P*. *falciparum* has only begun to be understood, as revealed in recent studies which observed programmed cell death at highly parasitized *in vitro* cultures^4–6^. However, the precise signal(s) that trigger the apoptosis-like events, remain to be elucidated.

During the last decade, there has been an increasing interest in extracellular vesicles (EVs) and their activity as carriers of a variety of signals, including those in protozoan parasites^7,8^. Several studies have shown the presence of circulating EVs from infected cells, which have been detected in infections caused by malaria parasites^9^. In 2013, two independent studies found that *P*. *falciparum* uses EVs for parasite-parasite and host-parasite communication, playing an important role in *in vitro* culture^10,11^. Further findings showed the participation of EV cargo in the host immune modulation, gametocytogenesis and gene regulatory functions^12–14^.

Previously, EVs had been identified as growth promoters or inhibitors in several cell lines, especially in cancer models (reviewed in^15^. Therefore, although there are no reports regarding EV activity as cellular suicide signals carriers in unicellular microorganisms, EVs could participate in quorum sensing (QS), as has been suggested in *Pseudomonas*, for biofilm formation and other social behaviors^16^. Interestingly, bacteria have the capacity of self-regulating their population density under stress through QS conditions, though there is not a direct relation with EVs^17,18^. Recently, the importance of the quorum sensing (QS) involvement in growth-density regulation and interspecies communication between trypanosomes was revealed^19^. Therefore, the potential use of QS in *P*. *falciparum* by an EV-based communication system that would induce programmed cell death (PCD) events as a social trait could explain parasite self-regulation.

On another aspect, *Pf*LDH, is one of the mayor markers used for malaria diagnosis^20^ and has been referred to as a possible target for treatment development^21^. The major function of *Pf*LDH is the conversion of pyruvate to lactate, a key process for energy production in most living organisms, producing lactate as a byproduct associated with the growth regulation in asexual stages of *P*. *falciparum*^22^. Interestingly, *Pf*LDH was previously identified as a component of EVs from *P*. *falciparum*^23^, which presented us with the possibility of this enzyme being involved in the control of parasite density through intercellular signaling. Therefore, in this study we focused on determining the activity of EV-associated *Pf*LDH as a means of regulation of *P*. *falciparum* density by stimulating apoptosis in highly parasitized cultures.

## Results

**Figure 1:**
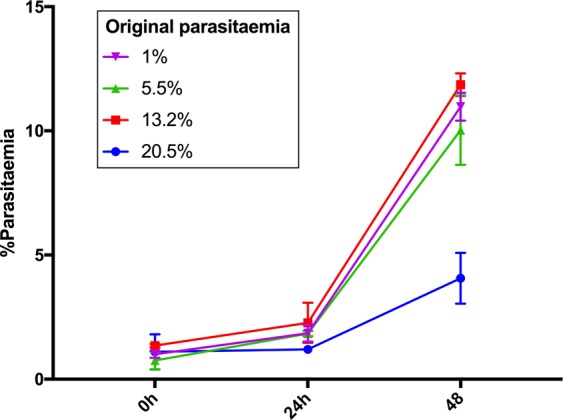
Highest parasitaemia growth. Cultures coming from 1%, 5.5%, 13.2% and 20.5% parasitaemias were split to 1% and monitored for 48 h.

We designed a growth bioassay to define the threshold of high parasitaemia of *P*. *falciparum in vitro* culture (Fig. 1). Cultures coming from different parasitaemias (13.2%, 5.5% and 1%) reached similar growth progression after all were split to 1% and monitored for 24 h. However, the culture with 20.5% parasitaemia decreased by half, even when it was split to 1% and received supplementary media and erythrocytes. Therefore, the criteria to define a low parasitaemia culture for the isolation of EV_L_ was the capability of continuous growth of 1–2% parasitized cultures. To define high parasitaemia cultures, we used parasitaemias of 20–25% without obvious morphological damage by light microscopy.

The size determination and characterization of the isolated *P*. *falciparum* EVs with the methodology used here was performed by our group in a previous study. For those experiments, 90% of EVs from high (EV_H_) and low (EV_L_) parasitized cultures showed the reported size of around 100 µm. In addition, we detected the enrichment of glycophorine A/B (GlyA), CD63 and PfMSP1, confirming their identity^24^. In this study, we labeled EV_H_ and EV_L_ with anti glycophorine and anti *Pf*LDH to compare the amount of *Pf*LDH in both populations. The flow cytometry analysis shows that 60% and 55% of the total EV_H_ and EV_L_, respectively, are associated with glycophorine. In this GlyA positive population, only 6.4% of EV_L_ contained *Pf*LDH. In contrast, 21.9% of EV_H_ were associated with *Pf*LDH (Supplementary Information 1).

**Figure 2:**
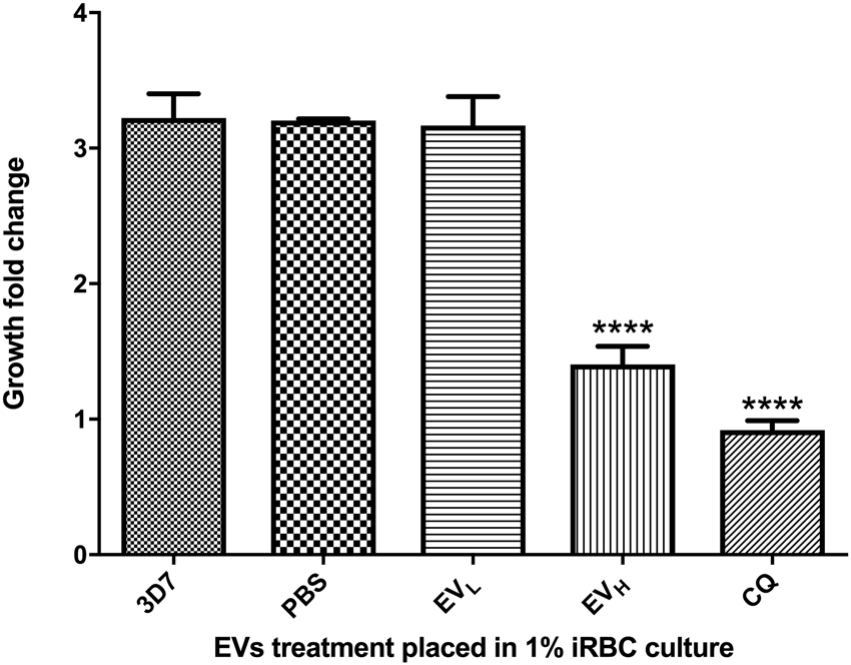
Growth inhibition of *P*. *falciparum* by EVs. The growth of parasites after distinct EV treatments was measured by flow cytometry. The average and S.D. of 3 replicas for each treatment is shown. (mean ± s.e.m.; n = 3 replicas), ****P < 0.0001 versus PBS and 3D7 growth control (Bonferroni’s test). Chloroquine^25^ was used as a drug control.

Flow cytometry analysis shows that EV_H_ that challenge the 1% schizont-targeted culture exert a significant reduction of growth after 24 h of incubation (Fig. 2). The EV_L_ and EVs from uninfected RBCs (EV_uRBC_) affected the targeted culture in minor scale when they are compared with the EV_H_ treatments. Therefore, for the following apoptosis marker comparisons, we used EV_L_ and EV_H_ isolated from cultures with 1–2% and 20–25% parasitaemias, respectively.

A characterization of the proteomic constitution of EVs from high or low parasite density cultures was performed to identify possible candidates that might be responsible for the inhibitory capacity of EV_H_. In total, we found 249 hits, of which 233 belong to *H*. *sapiens* and 16 to *P*. *falciparum* (Table 1). The comparative analysis used as criteria for the listing of a protein was that it appeared at least twice in one or both conditions. The 16 identified proteins of *P*. *falciparum* could not be associated exclusively to EV_H_ or EV_L_ samples because all of them were present in both conditions. However, changes in the relative abundance (expressed as Tfold in Table 1) show that *Pf*LDH is expressed more in EV_H_ samples by a Tfold of 2.6 (p < 0.05). In the case of human proteins, there are some differences in the proteomic composition after the comparison of EV_H_ with EV_L_. We found 9 protein hits (one of them a known contaminant) that were only present in EV_H_, which include F-box only protein 7, HSP90, IGLV5-45, Reelin, Thrombospondin-4, a desintegrin and metalloproteinase, and Talin-1. In the EV_L_ samples, 4 proteins were identified as only present in the condition of low parasitaemias.

**Table 1:** Differential proteomic analysis of EV_L_ and EV_H_ by nanoLC MALDI TOF TOF.

Name	Replica number	No. of total spectra	Description		
**Proteins in at least two replicas at only EV**_**L**_					
P04206	3	93	Ig kappa chain V-III region GOL OS = Homo sapiens		
J3KRP0	3	26	Beta-Ala-His dipeptidase OS = Homo sapiens		
Q5T985	2	1188	Inter-alpha-trypsin inhibitor heavy chain H2 OS = Homo sapiens		
A0A0B4J1U7	2	47	Protein IGHV6–1 (Fragment) OS = Homo sapiens		
Q5T4F6	2	17	Cartilage acidic protein 1 (Fragment) OS = Homo sapiens		
**Proteins in at least two replicas at only EV**_**H**_					
Q9Y3I1	2	9	F-box only protein 7 OS = Homo sapiens		
P98160	2	10	Basement membrane-specific heparan sulfate proteoglycan core protein OS = Homo sapiens		
P07900	2	12	Heat shock protein HSP 90-alpha OS = Homo sapiens		
A0A0G2JSC0	2	19	Protein IGLV5-45 (Fragment) OS = Homo sapiens		
P78509	2	10	Reelin OS = Homo sapiens		
P35443	2	19	Thrombospondin-4 OS = Homo sapiens		
Q76LX8	2	10	A disintegrin and metalloproteinase with thrombospondin motifs 13 OS = Homo sapiens		
Q9Y490	2	6	Talin-1 OS = Homo sapiens		
Name	Fold Change	pValue	Signal+	Signal−	Description
**Comparison of protein abundance levels in EV**_**H**_ ***vs*** **EV**_**L**_					
A0A024W2N3	−2.6	0.0094	31.5	12.3	L-lactate dehydrogenase OS = *Plasmodium falciparum*

**Figure 3:**
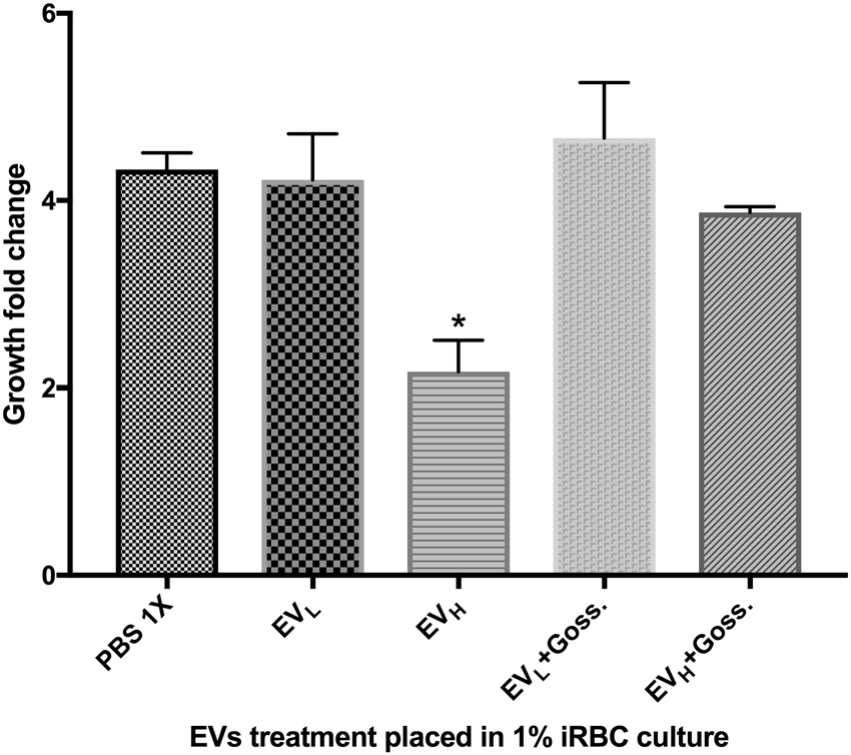
Inhibition of *Pf*LDH rescues the population regulation of EVs. Growth fold change was measured after distinct EV treatments and the addition of gossypol by flow cytometry. The average and S.D. of 3 replicas for each treatment is shown. (mean ± s.e.m.; n = 3 replicas), *P < 0.05 versus PBS control (Bonferroni’s test).

Based on the proteomic analysis, we decided to develop a series of bioassays to determine whether *Pf*LDH could be the inducer of the inhibition of parasite growth in high parasitaemia conditions. Thus, we designed a bioassay using gossypol, a reported inhibitor of *Pf*LDH. After determining a concentration that would not be detrimental to the parasites (Supplementary Information 2 and 3), we tested gossypol at 0.25 µM for its ability to reduce the inhibitory effect of EV_H_ in naive cultures. Remarkably, gossypol could rescue the parasite growth in naive cultures challenged with EV_H_, to the same growth level of untreated samples. Importantly, both EV_L_ and gossypol plus EV_L_ treatments did not significantly affect the growth of the targeted culture (Fig. 3).

**Figure 4:**
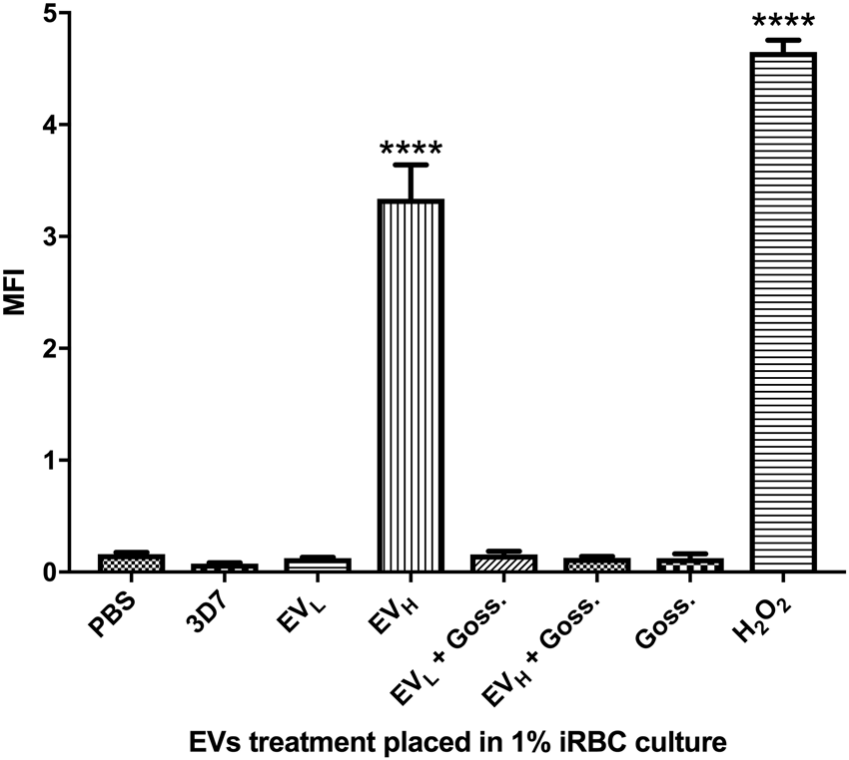
ROS detection after addition of EVs and 0.25 µM gossypol. MFI was measured by flow cytometry after diverse EV and gossypol treatment. The average and S.D. of 3 replicas for each treatment is shown. (mean ± s.e.m.; n = 3 replicas), ****P < 0.0001 versus PBS control (Bonferroni’s test).

We then evaluated whether the growth inhibition caused by EV_H_ could be linked to oxidative stress by measuring the levels of reactive oxygen species (ROS) after the addition of EV_H_ to a target culture. This experiment also included inhibiting *Pf*LDH and monitoring changes in the ROS levels. The results show that EV_H_ induced high levels of ROS in the target culture as soon as 4 h after incubation (Fig. 4), just as the positive control, 200 µM H_2_O_2_, did. Interestingly, this effect could be totally reverted when EV_H_ were also incubated with gossypol. Adding EV_L_ or gossypol by themselves had no impact on the production of ROS under the same conditions.

**Figure 5:**
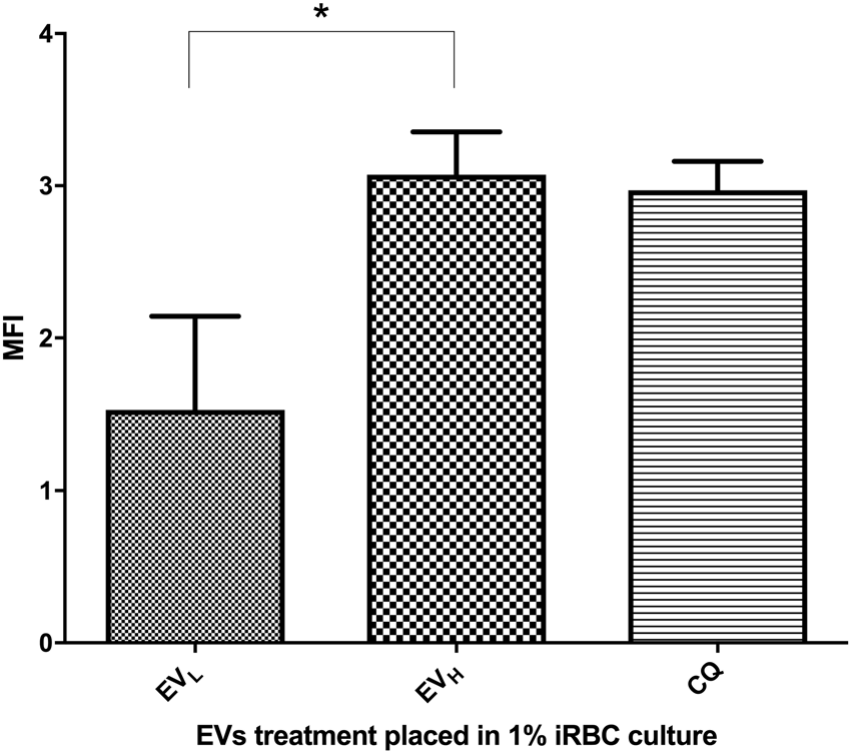
Phosphatidylserine translocation after challenge with EVs.

Several studies have shown that *P*. *falciparum* can present apoptosis markers under stress conditions^3,26,27^. Therefore, we proceeded to search for those markers in EV_H_-targeted cultures. First, we measured the externalization of PS on the membranes of iRBC after samples were incubated overnight with EV_H_. PS translocation levels in the targeted culture were significantly different from those elicited by treatment with EV_L_ (Fig. 5).

MFI was measured after diverse EV treatments by flow cytometry. The average and S.D. of 3 replicas for each treatment is shown. (mean ± s.e.m.; n = 3 replicas), *P < 0.05 EV_L_ versus EV_H_ (Bonferroni’s test).

**Figure 6:**
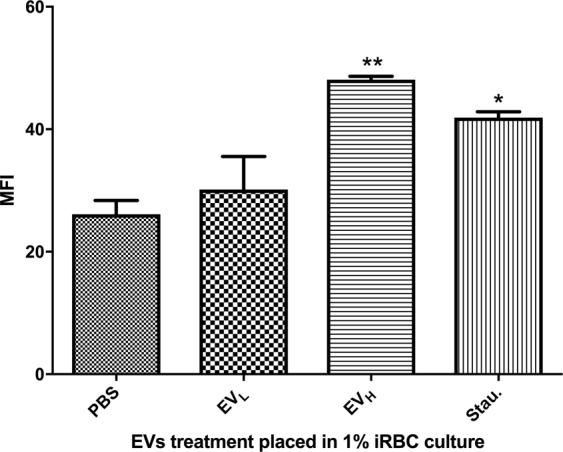
Caspase activity triggered by EV_H_. Caspase activity detection after EV treatment. MFI was measured after distinct EV treatments by flow cytometry. The average and S.D. of 3 replicas for each treatment is shown. (mean ± s.e.m.; n = 3 replicas), *P < 0.05, **P < 0.01, EV_L_ versus EV_H_ (Bonferroni’s test).

The increased activity of metacaspases has been reported previously as another indicator of the parasites commitment to apoptosis in *P*. *falciparum in vitro* cultures^26^. Therefore, we measured the caspase activation of the targeted cultures after being presented with EV_H_. We detected a significant increase in the level of pan-caspases when the infected erythrocytes were exposed to EV_H_ for 4 h, surpassing even those of the positive control, 10 µM staurosporine (Fig. 6). In contrast, there were no significant differences between the PBS 1X negative control and the challenge with EV_L_.

**Figure 7:**
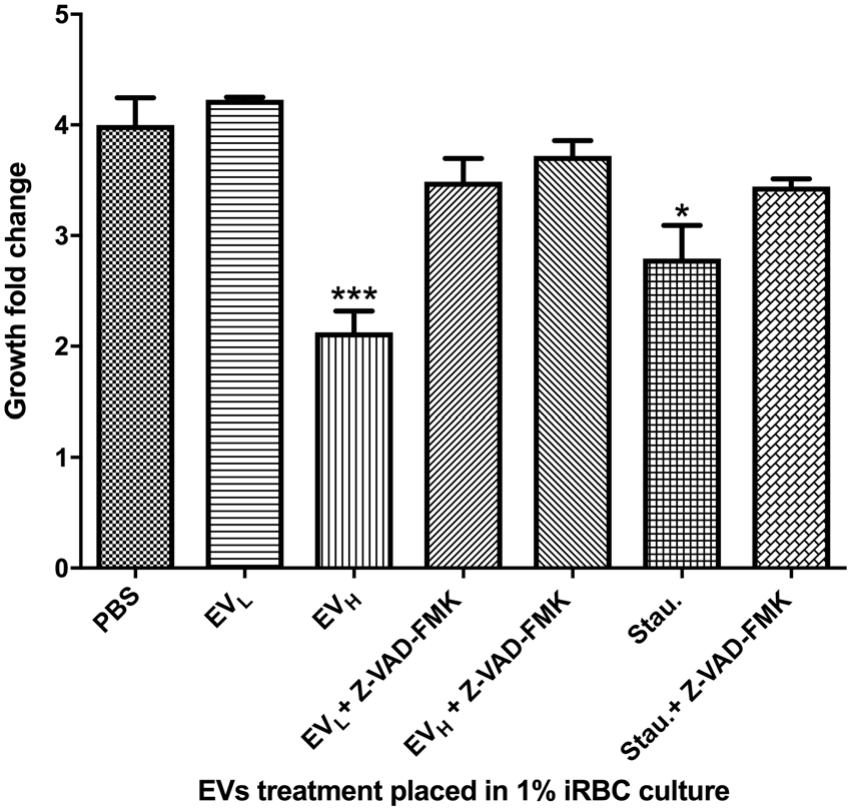
Rescued growth of *P*. *falciparum* after challenge with EV_H_ in the presence of a pancaspase inhibitor. Growth fold change was measured by flow cytometry after distinct EV treatments with the addition or not of the pancaspase inhibitor Z-VAD-FMK. The average and S.D. of 3 replicas for each treatment is shown. (mean ± s.e.m.; n = 3 replicas), ***P < 0.005 and *P < 0.05 versus PBS control (Bonferroni’s test).

To support these results, we tested the ability of the Z-VAD-FMK pan-caspase inhibitor to reverse the effects exerted by the addition of EV_H_ to target cultures. It is noteworthy stating that the inhibitory action of Z-VAD-FMK on metacaspases has been previously tested in *P*. *falciparum*, decreasing metacaspase induced-cell death without affecting the cell growth of controls, as decribed by Meslin *et al*.^28^ and Ch´ng *et al*.^27^. After 24 h, the growth of the samples challenged with EV_H_ but also treated with Z-VAD-FMK showed no significant differences with those where only PBS 1X had been used as control (Fig. 7). In comparison, the samples treated only with EV_H_ grew significantly less than unchallenged controls. As expected, the caspase activator, 10 µM staurosporine, reduced the parasites’ growth and this effect could be reverted when the pan-caspase inhibitor (10 uM) was present.

Finally, we performed a control experiment to discard necrosis as the main mechanism of death. We measured propidium iodide (PI) incorporation in the parasite nucleus, which is one of the main primary necrosis markers. In Supplementary Information 4, we show that EV treatment did not induce PI incorporation, which was only detected in those samples subjected to 80 °C for 5 minutes.

**Figure 8:**
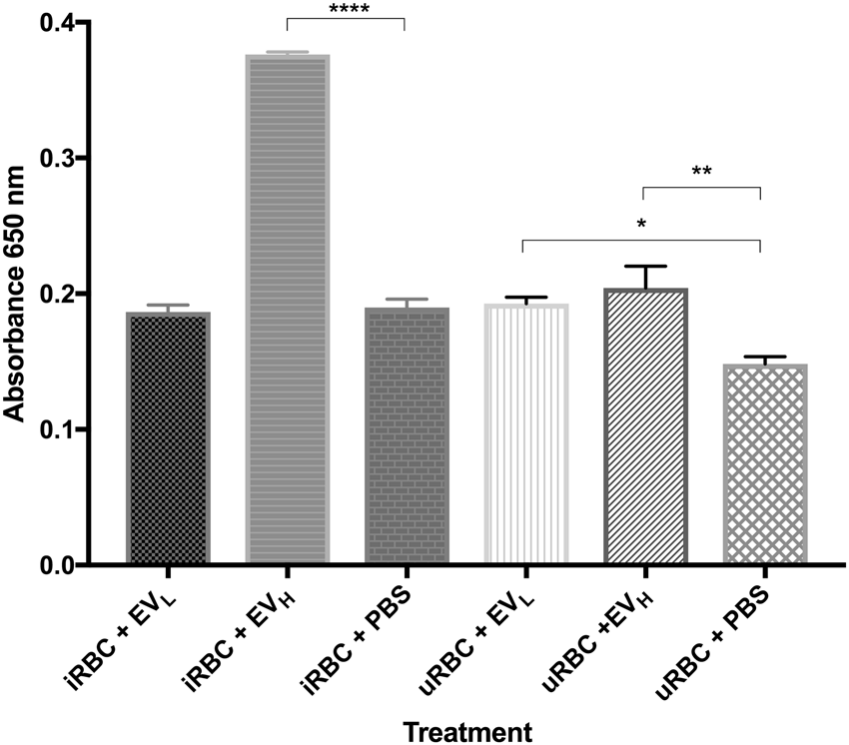
APAD bioassay to measure *Pf*LDH enzymatic activity caused by the addition of EVs. Absorbance was measured after distinct EV treatments at O. D. 650 nm. The average and S.D. of 4 replicas for each treatment is shown. (mean ± s.e.m.; n = 4 replicas), ****P < 0.0001 versus uRBC + PBS control (Bonferroni’s test).

To further prove the role of *Pf*LDH as a death-signaling molecule, we proceeded to measure the enzymatic activity of *Pf*LDH when iRBCs are exposed to EVs. To control for the contribution of LDH from the erythrocytes, uninfected RBCs were subjected to the same examination. Samples were analyzed 24 h after addition of EVs. Although there was a subtle increment in the activity of *Pf*LDH when iRBCs were incubated with EV_L_, it was insignificant. The addition of EV_H_, however, greatly boosted the activity levels of *Pf*LDH. Importantly, challenging uninfected RBCs with EV_H_ did not cause a major increase in *Pf*LDH activity (Fig. 8).

## Discussion

The hypothesis used in this study was based on the assumption that an uncontrolled proliferation of *P*. *falciparum* would lead to early death of the host, thus reducing the probability of transferring the infection to another vector and host^2^, and that to avoid this, the parasite would use means to control the density of its population.

To define what high parasitaemia means in *in vitro* culture, we first confirmed that death signals given off by density-stressed parasites became more growth-disruptive as the parasitaemia increased, and that the strength of these signals was such that even the addition of fresh RBCs and more nutrients at this point could not stop the instructions to die from being fulfilled. This commitment to die is accentuated when parasitaemias surpassed 20% in 3D7 *P*. *falciparum* cultures. Therefore, for this study we considered a parasitaemia of 20–25% to be high.

After differential proteomic analysis of the cargo of EVs isolated from low or highly parasitized cultures, the selected experiments were directed specifically to find the role of *Pf*LDH associated with EVs. *Pf*LDH has been studied for several decades due to its high expression during malaria infection. Taking advantage of its structural differences with human LDH, *Pf*LDH has been used for malaria diagnoses as well as in screening bioassays^29,30^. Although LDH has also been identified as a necrosis marker in mammals^31^, we have found evidence that *Pf*LDH could be involved in growth regulation via apoptosis.

The existence of a population stress in *P*. *falciparum* has been proposed previously, possibly by PCD, as a possible adaptation of the parasite to *in vitro* culture^32^. However, the authors offered no clues on what mechanisms caused the stress. In this study, we pondered two possible biological occurrences: (a) The incidental death of parasites due to a limiting source of nutrients and (b) the self-regulation of growth through communication between parasites. Based on the reports of the capability of EVs to carry resistance information to sensitive parasites, we studied the ability of EVs to be the regulators of the parasite population. The inhibition of growth of the parasites caused by challenging low-density cultures with EV_H_ gave us the first evidence that these vesicles could be associated with the death signal the *in vitro* culture was receiving. The lack of impact of a similar number of EV_L_ on their growth further confirmed this presumption.

Several assays were conducted to determine if the death signal sent the parasites on the way of apoptosis. That *P*. *falciparum* parasites can undergo a programmed cell death pathway was discussed recently when it was reported that highly parasitized *in vitro P*. *falciparum* culture displayed several programmed cell death processes such as DNA fragmentation, PS translocation and mitochondrial membrane depolarization. However, that study did not identify which factors trigger PCD under population stress^6^. After establishing that EVs from population-stressed cultures (EV_H_) can inhibit the growth of parasites in fresh cultures, we also found the common markers of apoptosis - augmented ROS levels, caspase activation and PS translocation - to be clearly present.

A proteomic analysis by nanoLC mass spectrometry of EVs from low or high-density parasitized cultures produced hits comparable to those found previously by^10^. This was especially true of the majority of the EV_L_ proteins. Nevertheless, we observed some differences when a differential scrutiny of EV_H_ and EV_L_ was performed_._ The comparative analysis of protein expression identified only *Pf*LDH as having increased EV_H_ in comparison with EV_L_. Interestingly, a recent study identified lactate as a possible growth regulator of *P*. *falciparum in vitro*^22^. Since lactate is the substrate of *Pf*LDH, we evaluated the impact of *Pf*LDH on the growth of *P*. *falciparum* and found it to be detrimental. Hampering the activity of EV_H_ with the *Pf*LDH inhibitor, gossypol, supported the proposed role of the *Pf*LDH in the regulation of growth when the number of iRBCs reaches critical levels. The activity state of the enzyme associated with EVs from *P*. *falciparum* was determined using the APAD bioassay, finding it increased in EV_H_.

ROS-increased levels indicate there is oxidative stress, which has been shown to be toxic to *P*. *falciparum*^33^. Although it has been suggested that ROS production is a host strategy to eliminate the parasite during infection through the action of phagocytes^34^, we have found that high levels of ROS can be produced in *P*. *falciparum* culture with only supplemented RBCs after addition of EV_H_. Remarkably, ROS levels could be totally reverted when gossypol was incubated alongside EV_H_, giving support to the hypothesis that high quantities of *Pf*LDH are the major stressing factor.

The identification of ROS was followed by the identification of caspase activation markers probably triggered by oxidative stress, already reported as related to apoptosis^35^. Despite *P*. *falciparum* having no classical caspases *per* se^36^, ancient analogue proteases fulfill similar biological functions and can be monitored by pan-caspase commercial kits^37^. Several publications have also identified that the triggering of metacaspases is involved in *P*. *falciparum* cell death, though identifying the complete machinery responsible for this process remains a pending assignment. For instance, it was reported that mefloquine, artemisinin derivatives and synthetic trioxane induce apoptotic cell death in plasmodium via ROS generation or a direct activation of the metacaspase enzyme^38,39^. Additionally, in a recent publication by Chou *et al*.^4^, it was shown that metacaspases are deregulated during high density culture and linked to an apoptotic-like mechanism. In addition, they found that the apoptosis-associated metacaspase gene Pfmc1 was upregulated significantly in this stress conditions. Other studies performed by Meslin *et al*.^26,28^ also find evidence of the induction of cell death by metacaspases which were inhibited with Z-VAD-FMK. In the bioassays performed by us, based on the measurement of these markers in the targeted culture, we detected the triggering of metacaspase after exposure to EV_H_. Confirmation of caspase activity was obtained upon the use of a pan-caspase inhibitor, which helped in the recovery of parasitaemia growth when parasites were challenged with EV_H_.

In a recent publication based in the hypothetical models of Chan and Bielski^40,41^ (by which superoxide triggers LDH to catalyze a free radical chain reaction), Hu *et al*.^42^ showed that human LDH in cancer cell lines can amplify ROS levels after the dominant anti-oxidative activity of LDH is switched to dominant pro-oxidative activity under oxidative stress. Hence, Hu *et al*. proposed that LDH could be a universal ROS amplifier in oxidative conditions by enhancing electron leakage from the electron transfer chain in the inner membrane of mitochondria where superoxide formation occurs, producing the amplification of ROS. Thus, in a similar fashion, *Pf*LDH can also be participating in the ROS amplification after EV_H_ are integrated with the target iRBCs at low parasitemia.

**Figure 9:**
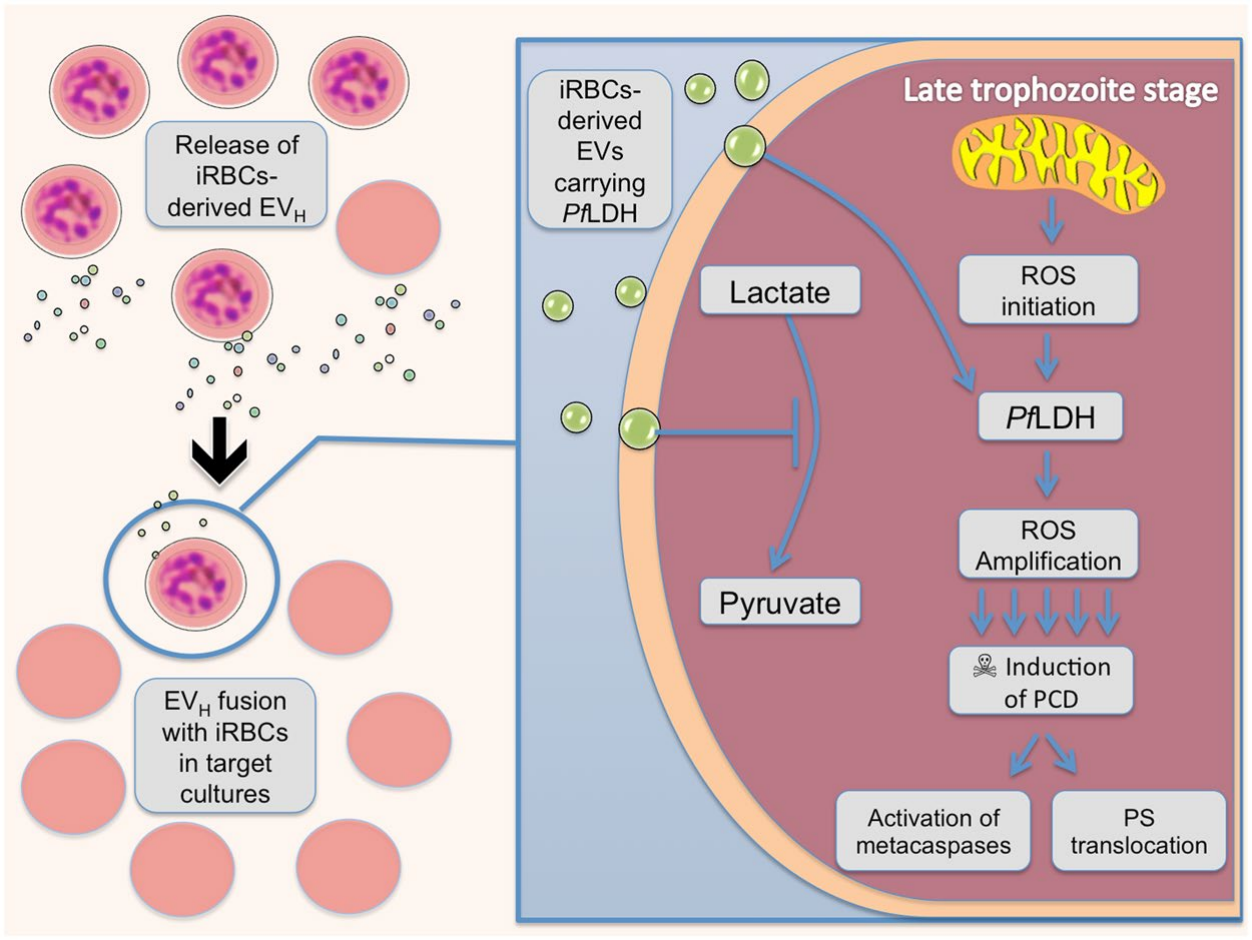
Diagram of the hypothesized action of *Pf*LDH in iRBCs after being transported by EVs. Density stressed cultures release EVs which cargo (PfLDH) is able to affect low density populations inducing death through apoptotic events triggered by this enzyme. PfLDH could inhibit the conversion of lactate to pyruvate producing an excess of the former which is detrimental to the parasite. Also, PfLDH could increment the production of ROS and the induction of apoptotic events leading to the suicide of the individual parasite.

The fact that EV_H_ by themselves do not show an incremented PfLDH activity when in presence of uninfected erythrocytes points to a factor in the iRBCs, that could well be the increased concentration in the metabolically highly active parasites of the LDH substrate pyruvate, that would be involved in augmenting the activity and subsequent conversion of this molecule to lactate. Our observations would be in accordance with those described by Hikosaka *et al*. (2015) that increased levels of lactate hinder the growth of *P*. *falciparum*. In any case, the demonstrated role of *Pf*LDH in regulating the number of parasites via EVs, which we present in this study, offers the first evidence that *P*. *falciparum* has the capacity to communicate a suicidal signal under population stress and that it does so through extracellular vesicles. A pictorial diagram of the proposed events is shown in Fig. 9.

## Methods

All reagents were from Sigma-Aldrich, Germany, unless specified.

### Ethical approval and informed consent

All experimental protocols were approved by the Bioethics Committee of the Gorgas Memorial Institute for Health Sciences. The methods were carried out in accordance with the relevant guidelines and regulations, and an informed consent was obtained from all volunteers that donated blood for cultures.

### *P*. *falciparum* culture

We cultured 3D7 *P*. *falciparum* (a kind gift of the Walter Reed Army Institute of Research, Silver Spring, MD, USA) using the conventional method of Trager & Jensen^43^ with modifications described in Almanza *et al*.^44^, that include the use of modified RPMI 1640 medium (Sigma-Aldrich, St. Louis, USA), 25 mM HEPES, 15 μM hipoxanthine, 50 mg/ml gentamicine sulfate, and 200 mM L-Glutamine, supplemented with 10% human serum, 2% sodium bicarbonate and a mix of gases (90% N_2_, 5% O_2_ and 5% CO_2_). Hematocrit was kept at 2%. Synchronization was performed in a temperature-cycling incubator (TCI) (Cooled Incubator, Model MIR-154, Sanyo, Japan) and by the addition of 0.3 M alanine (Sigma-Aldrich).

### Parasite highest growth stage

The highest parasitaemia that an *in vitro* culture of *P*. *falciparum* can reach before showing detrimental signs was determined by comparing the growth fold change between cultures with different parasitaemia. With this aim, 25 ml of iRBC at 5% parasitaemia contained in T75 flasks (Celltreat, China) were split or diluted with the corresponding addition of fresh RBCs into 5 cultures of 5 ml with different final parasitaemias: 1%, 2%, 4% and 5% in T25 flasks (Celltreat, China). After 48 h, cultures reached parasitaemias of 1%, 5%, 8%, 13% and 20% in schizont stage. At this point all cultures were diluted to approximately 1% through the addition of complete media and erythrocytes and monitored for 48 h by microscopy.

### EV isolation

EVs were obtained from 25 ml of alanine-synchronized infected RBCs (iRBCs) in late schizont stage or uninfected ones (uRBCs). Cultures with different parasitaemia (ranging from 2% to 25%) were used, depending on the conditions for each experiment. Parasitaemia was evaluated by optical light microscopy using Giemsa staining (GS500, Sigma-Aldrich). The isolation of EVs was based on previous reports^24,45^. Briefly, the cultures were collected and centrifuged at 2000 × *g* for 15 min. The 2000 × *g* supernatants were then centrifuged at 15,000 × *g* at 4 °C for 30 min to remove cell debris. Next, these supernatants were filtered through 0.2 μm low-binding protein filters (Acrodisc, Pall Life Science, Port Washington, USA) and the filtered supernatants were ultracentrifuged at 110, 000 × *g* 4 °C for 70 min to pellet small vesicles. The pellet was washed once by resuspending it in sterile double-filtered (0.2 μm) PBS 1X and further ultracentrifuged at 110,000 × *g* for an additional 70 min. The pellet was finally resuspended in 100 μl of double-filtered PBS 1× for analysis.

### EV characterization by Flow cytometry

The size-characterization of EVs by flow cytometry (CyFlow, Partec, Kent, UK) was performed using the same procedure and parameters reported by us in a previous study^24^. Data were acquired and analyzed using FloMax (Partec, Münster, Germany) software. Briefly, the EVs collected were classified by size using polyethylene beads of different denominations (0.1, 0.5 and 2 μm) (Fluka Analytical, Sigma-Aldrich). This was done before analyzing the samples as per other characteristics. The background noise per second was determined as the signal given off by double-filtered (0.2 μm) phosphate-buffered saline (PBS) solution. The final concentration of the samples was calculated using the software’s True Volumetric Absolute Counting tool, based directly on the basic definition of concentration c = N/V, using an electrode-principle determination.

### EV characterization

Isolated EVs were labeled with fluorescence antibodies to determine the presence of Glycophorin A coupled with Alexa 488 (Biolegend, California, United States) and *Pf*LDH (LS-C488831, Life Span Biosciencies, China), which was coupled with APC/Cy7 following the instructions of the conjugation kit (ab102859, Abcam, Cambridge, UK). We first filtered both primary and secondary antibodies with 0.2 µm filters to reduce possible false detection due to antibody aggregation. EV_L_ and EV_H_ were permeabilized with 0.05% saponin in PBS for 10 min. Then samples from cultures were incubated 3 h with primary antibodies at a dilution of 1:200 and then ultracentrifuged at 110,000 × g for 2 h. This last step was repeated to wash the EV pellet after which the supernatant was discarded. The pellet was resuspended in 100 µl of double-filtered PBS 1X and analyzed by flow cytometry.

## Bioassays

### Bioassay design and parasitaemia measurement

All the assays were performed in 96-well plates. After isolation, EVs were added immediately to an iRBC culture at 1% parasitaemia (targeted culture). Flow Cytometry was used to measure parasitaemia by staining iRBC with 2 µg/ml Hoechst 33342 using the methodology reported by Coronado *et al*.^46^.

### EV action on naive cultures

We determined the growth inhibition exerted by EVs isolated from high and low parasitaemia cultures by adding EVs at 2 × 10^5^ particles/ml into 2% parasitized naive cultures in mature trophozoite stages. Cultures were then incubated for 24 h and the new parasitaemia was measured by flow cytometry. The inhibitory activity of EVs was determined from measuring fold changes of parasitaemia in the cultures. We used chloroquine 10 µM as positive control for death and apoptosis.

### Proteomic analysis

Differential analysis of the proteomic content of EVs from low and high parasitaemia cultures was performed using nano LC mass spectrometry (4800 MALDI TOF/TOF Mass Spectrometer, Abi Sciex, Foster City, CA, USA). Briefly, after resuspension of EVs with 100 µl of lysis buffer (20 mM Tris, 7 M Urea, 2 M Thiourea, 4% CHAPS 2 mM EDTA, 1 mM PMSF, 2% Triton X-100, 1 µl Protease Inhibitor cocktail and 10 mM DTT, all from Sigma Aldrich). After this, 20 µg of EV lysates were run in one-dimensional PAGE (Invitrogen, Carlsbad, CA, USA) in triplicates and then stained with SilverQuick (Invitrogen). Each PAGE lane was cut in six parts and unstained with 30 mM potassium ferricyanide and 100 mM sodium thiosulfate before incubating them with 10 mM DTT at 56 °C for 1 h, followed by 55 mM IAA at 30 °C for 45 min. Next, samples were digested with 0.5 µg of trypsin (Sequencing Grade Modified Trypsin, Promega, Southampton, UK) and 50 mM BCA at 37 °C overnight. Peptides were extracted from gels using 60% ACN/0.1% TFA; solvents were evaporated by speed-vac. Next, samples were resuspended with H2O/0.1% TFA and then cleaned with microcolumns C18 (ZipTip Pipette Tips, Millipore, Billerica, MA, USA). Samples were dried by speed-vac and resuspended again with H2O/0.1% TFA. After this step, samples were loaded in the nanoLC system (EASY-nLC 1000, Thermo Scientific, San Jose, CA, USA) using an Easy column 50 cm Å~75 μm ID, PepMap RSLC C18, 2 μm (Thermo Scientific). Mobile phases were (A) H2O/0.1% TFA and (B) ACN/0.1% TFA, in a B gradient of 0–50% during 100 min with a flux of 250 nL/min. Peptides were detected in-line by a mass spectrometer LTQ Velos (Thermo Scientific) using a data-dependent gaining protocol supported by a dynamic exclusion list. The data were analyzed using a database generated with the software Pattern Lab for Proteomics (Carvalho *et al*., 2012 and Carvalho *et al*., 2016) and then compared with the Uniprot sequence database (www.uniprot.org) of *P*. *falciparum* and *H*. *sapiens*. Proteomic standard controls were included in the database to discard any contaminant in the experiment. Search parameters were set to include the variable modification of methionine oxidation and the fixed modification of cysteine carbamidomethylation at tolerance 800 ppm for m/z precursor. The PSMs were filtered using SEPro from Pattern lab for proteomics and an FDR cutoff of 1% for protein level and a minimum of two peptides for protein.

### Phosphatidylserine translocation

PS translocation was detected with the Annexin V-FITC apoptosis detection kit II (BD Pharmingen, Heidelberg, Germany). Briefly, aliquots of the challenged cultures were collected after overnight incubation with the added EVs. Samples were washed with PBS and resuspended in 1X binding buffer with 5 µl of Annexin V-FITC for 15 min at RT. Flow Cytometry with the 488 nm argon laser was used to analyze the samples. Erythrocytes were gated on a forward- *versus* side-scatter dot blot and pRBC were discriminated on a FL 5 integral^47^ histogram for Hoescht fluorescence. Gated pRBC were analysed for annexin V-FITC fluorescence on a FL1 integral^47^ histogram (Supplementary Information 5). At least 20 000 events in the erythrocyte gate were counted.

### Caspase activation

Caspase activation was measured with the Pan-Caspase detection kit (Millipore Merck, Germany). For this experiment, 100 µl of each sample were washed once and resuspended in 300 µl of PBS 1 × . FLICA 1X was added and the sample incubated for 1 h at 37 °C, after which they were washed once with the wash buffer and resuspended with 750 µl of PBS 1×. The level of fluorescein in the samples was analyzed by flow cytometry with the 488 nm argon laser. In addition, we used the pan-caspases inhibitor 10 µM Z-VAD-FMK (Abcam, Cambridge, UK) to confirm the specificity of the results.

### Reactive oxygen species (ROS)

The level of ROS in the cultures challenged with EVs was performed using CM-H2DCFDA (Thermofisher Scientific, San Jose, CA, US). Samples were transferred to 1.5 ml microcentrifuge tubes and centrifuged at 1,000 × *g* for 1 min and washed once with PBS 1×. Samples were resuspended in RPMI 1640 and incubated for 30 minutes at 37 °C. As positive control, 100 μM hydrogen peroxide was used. The intracellular ROS production was measured by flow cytometry with at 488 nm argon laser as light source and the green fluorescence emission at 517 measured in the FL1 channel.

### APAD Bioassay

The assay to measure the activity of *Pf*LDH is based on the reduction of 3-acetyl pyridine adenine dinucleotide (APAD) to APADH, a NAD+ derivative that is specific for *Pf*LDH, which allows for the distinction of *Pf*LDH from that of the host RBC (Makler *et al*., 1993). Using a modified protocol of D’Alessandro *et al*. (2013), we measured the *Pf*LDH activity in EV-challenged iRBCs as well as in samples composed uniquely of EVs and PBS. Due to the limited volume of EV samples, readings were taken only at 0 and 24 h. The formation of APADH was measured at 650 nm in a multiplate reader (Synergy HT, Biotek, Winooski, VT, USA) using a solution containing 1 mM phenazine ethosulfate, 2 mM nitroblue tetrazolium, 12.5 mM lactate, and 50 mM APAD+ in PET buffer consisting of 50 mM Tris–HCl, 1 mM EDTA and 0.01% Triton X-100 at pH 9.0.

### Statistical analysis

Data from different experiments examining flow cytometer or fluorometer measurements in response to EV treatment are presented as mean ± standard error (SE) of three individual samples per variable. All experiments were independently repeated at least twice, and the mean results of all experiments are presented. Data were evaluated by analysis of variance, and significant differences between groups were determined using Bonferroni’s test. A p ≤ 0.05 was considered significant.

## Supplementary information

Supplementary Information
