# Author Correction: Extracellular vesicles carrying lactate dehydrogenase induce suicide in increased population density of *Plasmodium falciparum in vitro*

**DOI:** 10.1038/s41598-020-69582-y

**Published:** 2020-07-27

**Authors:** Ricardo Correa, Lorena Coronado, Zuleima Caballero, Paula Faral-Tello, Carlos Robello, Carmenza Spadafora

**Affiliations:** 1 Center of Cellular and Molecular Biology of Diseases, Instituto de Investigaciones Científicas y Servicios de Alta Tecnología (INDICASAT AIP). City of Knowledge, Panama City, 0843-01103 Panama; 2 Department of Biotechnology, Acharya Nagarjuna University, Guntur, 522 510 A.P. India; 3 Institut Pasteur, Montevideo, Uruguay; 4 Sistema Nacional de Investigación, Secretaría Nacional de Ciencia, Tecnología e Innovación, Panama City, 0843-01103 Panama

Correction to: *Scientific Reports* 10.1038/s41598-020-69582-y, published online 25 March 2019

The original version of this Article contained a typographical error in the spelling of the author Paula Faral-Tello, which was incorrectly given as Paula Faral.

As a result, the author contribution statement was incorrect:

“L.M.C. and Z.C. contributed to sample preparation and experiments. P.F. helped with, and C.R. was responsible for, the proteomic data analysis. C.S. conceived and was responsible for the study and R.C. developed and enhanced it, performing most of the experiments, including sample preparation for proteomics, and took the lead in writing the manuscript in consultation with C.S. The latter was reviewed by all authors with critical feedback with final revision and approval by C.S.”

now reads:

“L.M.C. and Z.C. contributed to sample preparation and experiments. P.F-T. helped with, and C.R. was responsible for, the proteomic data analysis. C.S. conceived and was responsible for the study and R.C. developed and enhanced it, performing most of the experiments, including sample preparation for proteomics, and took the lead in writing the manuscript in consultation with C.S. The latter was reviewed by all authors with critical feedback with final revision and approval by C.S.”

These errors have now been corrected in the PDF and HTML versions of the Article, and in the accompanying Supplementary Information file.

