# Supplementary material for: Extracellular vesicles carrying lactate dehydrogenase induce suicide in increased population density of Plasmodium falciparum in vitro
Source: Sci Rep. 2019 Mar 25;9:5042. doi: 10.1038/s41598-019-41697-x (PMC6434017; doi:10.1038/s41598-019-41697-x)
Supplement: Supplementary file 1 — Supplementary Information [file 41598_2019_41697_MOESM1_ESM.pdf]

Extracellular vesicles carrying lactate dehydrogenase induce suicide in increased population density of *Plasmodium falciparum in vitro*

Ricardo Correa, Lorena Coronado, Zuleima Caballero, Paula Faral, Carlos Robello, Carmenza Spadafora

SUPPLEMENTARY INFORMATION

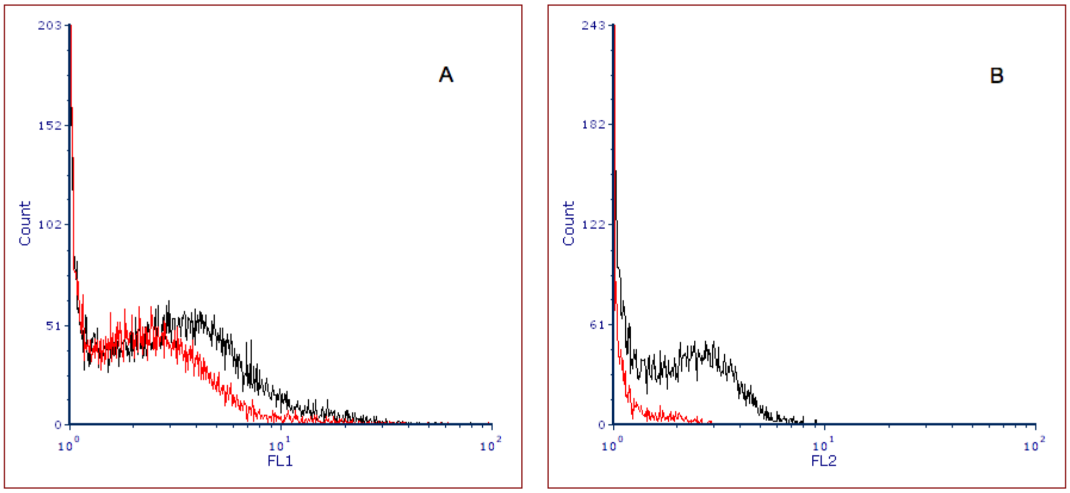

Supplementary Information 1. Characterization of EVs by flow cytometry. Red and black lines correspond to EV_L and EV_H, respectively. In (A), the association of GlyA with EVs was measured with Alexa 488 (FL1). In (B), *Pf*LDH association with EVs of the GlyA-positive population was measured with APC/C7 (FL2).

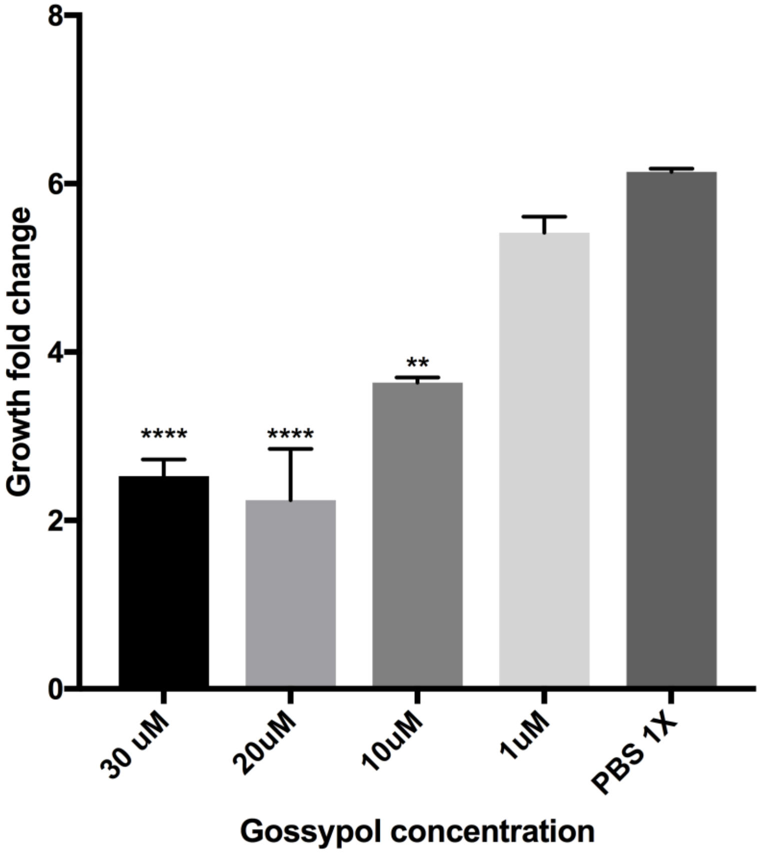

Supplementary Information 2. Lethal dose concentration of gossypol. The growth of parasites after distinct gossypol concentration was measured by flow cytometry. The average and S.D. of 3 replicas for each treatment is shown. (mean ± s.e.m.; n = 3 replicas), ****P < 0.0001 and **P< 0.01 versus PBS control (Bonferroni's test).

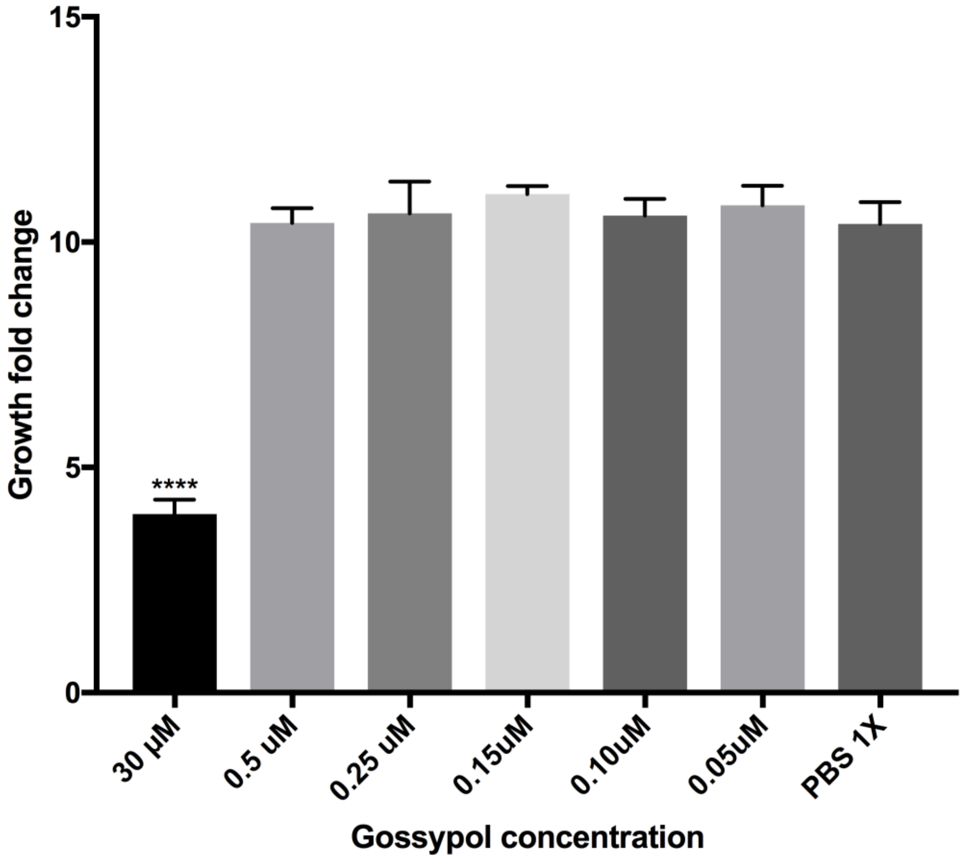

Supplementary Information 3. **Safety dose concentration of gossypol.** The growth of parasites after distinct gossypol concentration was measured by flow cytometry. The average and S.D. of 3 replicas for each treatment is shown. (mean ± s.e.m.; n = 3 replicas), ****P < 0.0001 versus PBS control (Bonferroni’s test).

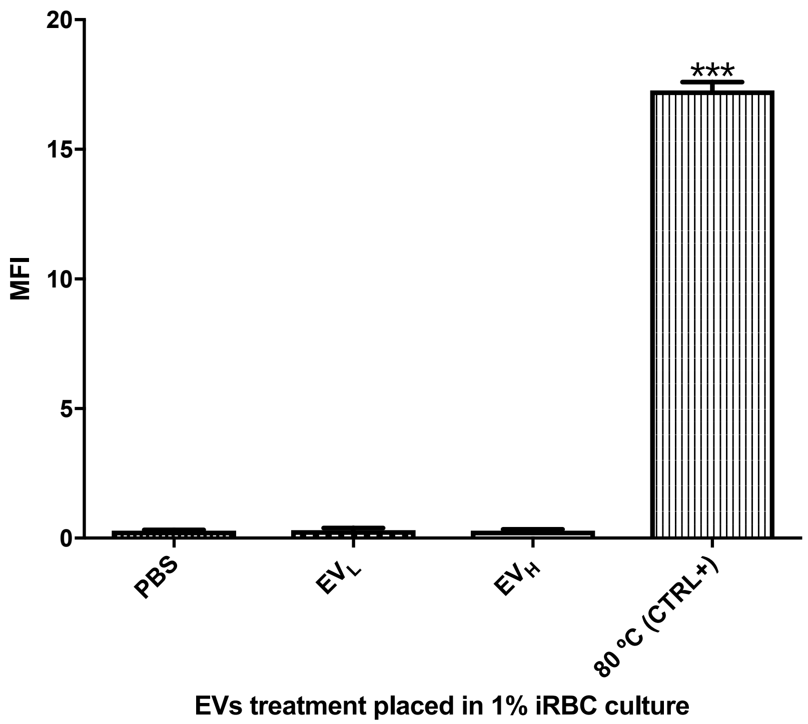

Supplementary Information 4. Assessment of necrosis in EV-challenged *P. falciparum* cultures. MFI was measured after distinct EV treatments or heat (80 ºC for 30 min) by flow cytometry. The average and S.D. of 3 replicas for each treatment is shown. (mean ± s.e.m.; n = 3 replicas), ***P < 0.005 versus PBS control (Bonferroni's test).

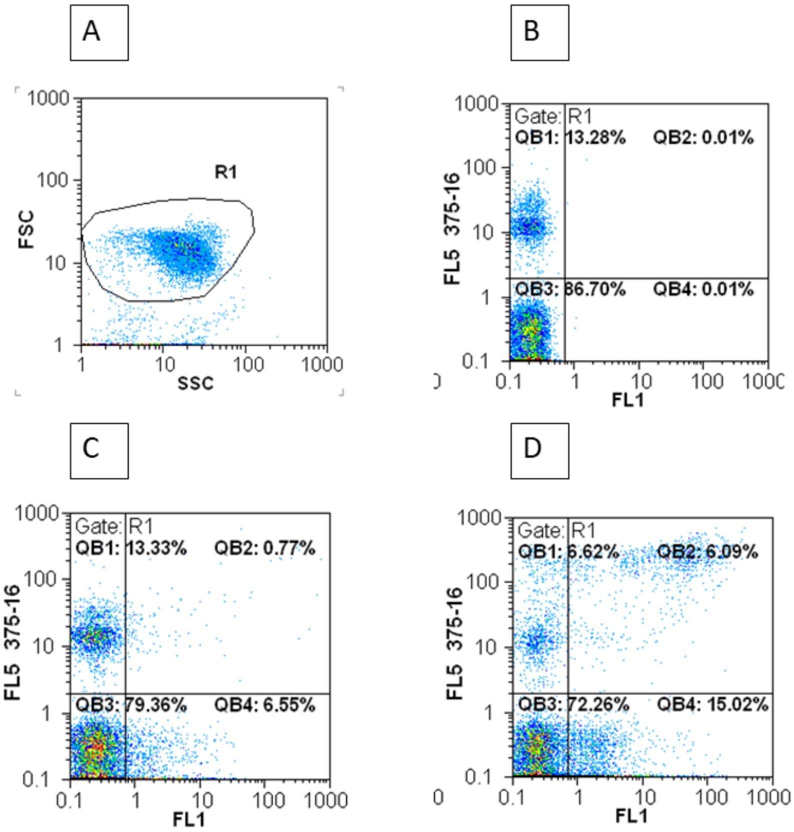

Supplementary Information 5: **Representative dot plots of phosphatidylserine translocation experiments**. The gating on iRBC was done with the positive population for FL5 (Hoescht staining) (QB1) which was analyzed for FL1 signal (Annexin V) (QB2). FL5 was plotted against FL1 to get the positive percentage of all populations. A. FSC vs SSC dot plot showing the population B. Negative control (Uninfected erythrocytes) C. Low parasitemia EVs D. High parasitemia EVs.
